# Frequency of Intraoperative Hypotension After the Induction of Anesthesia in Hypertensive Patients with Preoperative Angiotensin-converting Enzyme Inhibitors

**DOI:** 10.7759/cureus.6614

**Published:** 2020-01-09

**Authors:** Fahad Salim, Fazal Khan, Muhammad Nasir, Rashid Ali, Ayesha Iqbal, Amir Raza

**Affiliations:** 1 Anaesthesiology, Aga Khan University, Karachi, PAK; 2 Chest Medicine, Jinnah Postgraduate Medical Centre, Karachi, PAK; 3 Oral Pathology, Sir Syed Institute of Medical Sciences, Karachi, PAK

**Keywords:** hypotension, hypertension, anesthesia, angiotensin converting enzyme inhibitors, ace inhibitors

## Abstract

Introduction

The renin-angiotensin-aldosterone system (RAAS) is an important target in the treatment of hypertension. Angiotensin-converting enzyme (ACE) inhibitors block the conversion of angiotensin I to angiotensin II. ACE inhibitors not only treat hypertension but also decrease morbidity and mortality in heart failure patients and in patients with acute myocardial infarction. The discontinuation of ACE inhibitors before the surgery is still controversial. To assess the current magnitude of the problem in our population, we aimed to conduct this study, which evaluated the frequency of intraoperative hypotension after the induction of anesthesia in controlled hypertensive patients with preoperative ACE inhibitors.

Material and methods

This descriptive case series study was conducted at a tertiary hospital in a developing country after approval from the Ethics Review Committee. A total of 115 adult patients, from 16 to 60 years of age, who have undergone elective surgery, have controlled hypertension on the desired drugs for at least six months, have no history of any cardiac event, and have taken the drug on the morning of the surgery, were included in the study after written consent. The demographic data of the patients were entered into the proforma. Preoperative systolic, diastolic, and mean arterial pressure were recorded by the researcher or an assignee in the preoperative holding area. The patients were followed in the recovery room by the team conducting the study until 10 minutes after the arrival of the patient in the recovery room. All statistical analyses were performed using Statistical Packages for the Social Sciences version 19 (SPSS Inc., Chicago, IL). p-value ≤0.05 was considered significant.

Results

Of the 115 patients, 56 (48.7%) patients were in the age group between 51 and 60 years of age; 38 patients were between the ages of 41 and 50 years and only 21 patients were 40 years or less. On gender, 68 patients were female and 47 were male. According to body mass index (BMI), the majority of the patients were in the overweight group, amounting to 53 (46%), and 86 (74.78%) patients were known diabetics. Overall, 77 (66.96%) of the patients developed intraoperative hypotension with 41 (35.65%) patients requiring the use of vasopressors in order to correct the hypotension. No statistically significant difference was found between demographic and clinical variables.

Conclusion

Intraoperative hypotension is more frequent in patients with controlled hypertension on ACE inhibitors although more studies need to be conducted on a larger population in order to determine a more definitive result.

## Introduction

The renin-angiotensin-aldosterone system (RAAS) is an important target in the treatment of hypertension. Angiotensin II production is the final common pathway in the renin-angiotensin-aldosterone system. Angiotensin-converting enzyme (ACE) inhibitors block the conversion of angiotensin I to angiotensin II [1]. ACE inhibitors not only treat hypertension but also decrease morbidity and mortality in heart failure patients and in patients with acute myocardial infarction. This class of drug is well-indicated in patients for the treatment of hypertension. Many studies recommended that beta-blockers and alpha 2 agonists should be continued till the operative day, as their discontinuation may cause more harm than benefit.

Studies regarding ACE inhibitor use are far more conflicting in nature [2-4]. In the past two decades, numerous studies have been done on the use of ACE inhibitors preoperatively [5]. Drenger, in his study on the use of ACE inhibitors in patients undergoing coronary artery bypass grafting (CABG), found that the withdrawal of ACE inhibitors after CABG was associated with nonfatal in-hospital ischemic events. Also, studies such as the one done by Schulte on 36 patients to study the effect of long-term ACE inhibitor concluded that long-term ACE inhibitor use does not aggravate a decrease in blood pressure in minor surgeries [6]. Both authors suggest that the discontinuation of ACE inhibitors doesn’t give an additional benefit. In contrast, Nabbi R et al. presented a case report of a 51-year-old female who underwent general anesthesia and developed hypotension on induction despite the discontinuation of the medicine 24 hours prior to the surgery and concluded that angiotensin II receptor blockers (ARBs) along with ACE inhibitors should be withheld 24 hours prior to surgery and possibly longer to elude unnecessary morbidity and mortality [7]. Ryckwaert F et al. and Smith I et al., in their paper ‘Changing concepts in anesthesia for daycare surgery,' stated that continuing ACE inhibitors and angiotensin receptor blockers may increase the likelihood of intraoperative hypotension but it will respond to simple treatments without any apparent adverse outcomes [8-9]. Coriat P et al. did a study on 51 patients who continued their ACE inhibitor therapy till the morning of the surgery and concluded that in hypertensive patients chronically treated with ACE inhibitors, maintenance of therapy until the day of surgery may increase the probability of hypotension at induction [10]. One of the most recent studies was conducted by Khan et al. on 92 patients; it showed that 55 patients developed hypotension within 30 minutes of the beginning of surgery; however, they concluded that more studies were needed to be done on a larger population [11]. The rationale of the current study was to assess the current magnitude of the problem in our population; if the results of this study show an increased frequency of intraoperative hypotension, suggestions will be recommended accordingly in the future to manage such complications. So the objective of our study was determining the frequency of intraoperative hypotension after the induction of anesthesia in controlled hypertensive patients with preoperative ACE inhibitors.

## Materials and methods

This descriptive case series study was conducted at a tertiary hospital in a developing country after approval from the Ethics Review Committee. The calculation of sample size was based on a previous study. Khan MA et al. reported that after the induction of anesthesia, at 30 minutes, hypotension was observed in 59.8% cases so p=59.8% and margin of error=9%; therefore, at least 115 patients were included in this study [11]. The non-probability consecutive sampling technique was used. All adult patients from 16 to 60 years who had undergone elective surgery, with controlled hypertension on the desired drugs for at least six months, with no history of any cardiac event, and those who had taken the drug on the morning of the surgery were included in the study. Those with hypotension (systolic blood pressure < 90) at the time of preoperative evaluation, uncontrolled hypertension (systolic blood pressure > 150 or diastolic blood pressure > 95) at the time of preoperative evaluation, surgery for pathology related to vasoactive substances (carcinoid, pheochromocytoma), left ventricular ejection fraction less than 40%, clinical evidence of decompensated heart failure at the time of preoperative evaluation, and end-stage renal disease were excluded.

Preoperative evaluation was done to assess the patient according to the inclusion and exclusion criteria. Written consent was taken after informing the patient about the study. Upon receiving consent, the demographics data of the patient were entered into the proforma. The primary anesthetist and surgical team were informed about the study. Preoperative systolic, diastolic, and mean arterial pressure were recorded by the researcher or an assignee in the preop holding area. The intraoperative monitoring chart was maintained by the primary anesthesia team and was followed by the research team. The patient was followed in the recovery room by the team conducting the study until 10 minutes after the arrival of the patient in the recovery room. As there is no protocol of withholding ACE inhibitors in our institution, it is understood that the patient had taken the drug prior to surgery so there was no blinding of any sort, however, the primary anesthesia team was informed about the study by the research team. Standard monitoring was done intra and postoperatively (noninvasive blood pressure, oxygen saturation, heart rate, and body temperature). If hypotension developed, it would be at the discretion of the primary anesthesia team when and whether to correct it with the use of intraoperative fluid administration or vasopressor support as they deemed fit and the research team would not interfere with their decisions as long as all the interventions are recorded without fail. The vitals were recorded at an interval of 10 minutes intraoperatively by the primary team and in the recovery room by the recovery room staff.

Data analysis procedure

All statistical analysis was performed using Statistical Packages for Social Sciences version 19 (SPSS Inc., Chicago, IL). Frequency and percentage were computed for qualitative observations like gender, American Society of Anesthesiologist (ASA) status, and hypotension. Mean and standard deviation were estimated for age, body mass index (BMI), systolic blood pressure (BP). Stratification was done to controlled effect modifiers like age, gender, BMI, and diabetes to observe the rate of hypotension. The chi-square test was used to observe the modifiers' effect on hypotension. p≤0.05 was considered statistically significant.

## Results

Overall, 115 patients were included in the study based on the inclusion and exclusion criteria. Of the 115 patients, 56 (48.7%) patients were in the age group between 51 and 60 years old. A total of 38 patients were between 41 and 50 years and only 21 patients were 40 years or less of age. Descriptive statistics of age and duration of anesthesia are described in Table 1.

**Table 1:** Descriptive statistics of age and duration of anesthesia

Statistics	Age (Years)	Duration of Anesthesia (min)
Mean ± SD	49.02 ± 8.69	76.61 ± 33.08
95% Confidence Interval for Mean Lower Bound - Upper Bound	47.41-50.62	70.5 – 82.72
Median (IQR)	50(12)	70(30)
Minimum	19	20
Maximum	60	200

Out of 115 patients, 68 were female and 47 were male. According to BMI, the majority of the patients were in the overweight group, amounting to 53 (46%), followed by the group with normal BMI (47 (40%)) with the obese and morbid obese group comprising 10 (8.7%) and 5 (4.35%), respectively. A total of 103 patients were labeled as ASA II and 12 patients were labeled as ASA III due to either uncontrolled diabetes or morbid obesity. There were no ASA IV patients. Of the 115 patients, 86 (74.78%) patients were known diabetics. Although all the patients were on ACE inhibitors, 31 (29.96%) patients were also on some other antihypertensives; mostly, these drugs were either diuretics or calcium channel blockers. Intraoperative hypotension was observed in 77 (66.96%) patients in which 41 (35.65%) patients required the use of vasopressors to correct the hypotension. A higher frequency of hypotension was noted in the age group between 41 and 50 years of age (73.7%) although the difference was not statistically significant, as shown in Table 2.

**Table 2:** Frequency of intraoperative hypotension after the induction of anesthesia in controlled hypertensive patients with preoperative ACE inhibitors with respect to demographic variables ACE: angiotensin-converting enzyme

Variables	Hypotension		Total	p-value
	Yes	No		
Age Groups (Years)				
≤ 40	10(47.6%)	11(52.4%)	21	0.105
41 to 50	28(73.7%)	10(26.3%)	38	
>50	39(69.6%)	17(30.4%)	56	
Gender				
Male	30(63.8%)	17(36.2%)	47	0.55
Female	47(69.1%)	21(30.9%)	68	
BMI				
Normal	29(61.7%)	18(38.3%)	47	0.57
Overweight	39(73.6%)	14(26.4%)	53	
Obese Morbidly	6(60%)	4(40%)	10	
Obese	3(60%)	2(40%)	5	

No differences were found between clinical variables like diabetes or ASA status that were statistically insignificant, as tabulated in Table 3.

**Table 3:** Frequency of intraoperative hypotension after induction of anesthesia in controlled hypertensive patients with preoperative ACE inhibitors with respect to clinical variables ACE: angiotensin-converting enzyme

Variables	Hypotension		Total	P-value
	Yes	No		
Diabetic				
Yes	19(65.5%)	10(34.5%)	29	0.84
No	58(67.4%)	28(32.6%)	86	
ASA-Status				
ASA-II	69(67%)	34(33%)	103	0.98
ASA-III	8(66.7%)	4(33.3%)	12	
Patient on other antihypertensive drugs other than ACE Inhibitor				
Yes	22(71%)	9(29%)	31	0.57
No	55(65.5%)	29(34.5%)	84	

## Discussion

There have been numerous studies done on the continuous use of ACE inhibitors until the day of surgery. Although there have been discussions on different forums, it is still a topic of debate whether the use of ACE inhibitors preoperatively is justified or not. Some researchers recommend that it is reasonable to continue ACE inhibitor perioperatively, while some also recommended that if the drug is held preoperatively, it should be started as soon as clinically feasible [12]. There are conflicting views in regard to the effects of ACE inhibitors on intraoperative hemodynamics. As Behnia et al. state, chronic ACE inhibitor therapy does cause an increased incidence of hypotension intraoperatively. There are also studies of contrasting opinions like the one done by Pigot et al., which addresses the profound postoperative hypertension in patients who withheld ACE inhibitor before surgery, which can lead to detrimental effects [13-14]. Hence, the discussion on whether ACE inhibitor therapy should be continued preoperatively is left unanswered.

In this observational study, we enlisted 115 patients who were on ACE inhibitors, according to the inclusion and exclusion criteria set at the commencement of the study. The aim of this study was to assess the frequency of intraoperative hypotension in patients who had controlled hypertension on ACE inhibitor therapy. The variables set prior to the study were age, gender, BMI, ASA status, diabetes, and if the patients were on any other antihypertensive in addition to ACE inhibitors and their associations with intraoperative hypotension. It was observed in the end that 77 patients out of 115 (66.96%) did develop hypotension within 30 minutes of induction of general anesthesia. These results are comparable to the study done by Khan et al. in 2012 that had enlisted 92 patients in their study and showed that 59.8% of the patients had hypotension at 30 minutes and 40.2% of the patients had hypotension at 60 minutes of the induction of anesthesia [11]. Khan et al. then further divided hypotension into mild, moderate, and severe hypotension although, in our study, the criteria were set at systolic blood pressure falling below 90 mmHg within 30 minutes of the induction [11]. Similar results were posted by Comfere et al. who evaluated a total of 267 seven patients receiving chronic antihypertensive treatment with ACE inhibitor and undergoing noncardiac surgery under general anesthesia [15]. The results showed that the patients who had taken the drug 10 hours before the surgery were more prone to intraoperative hypotension within the first 30 minutes of the surgery. They concluded that ACE inhibitor therapy should be ceased at least 10 hours before surgery. A randomized, prospective, double-blinded study was conducted by Rajgopal in 2014, which included 60 patients, and the aim was to determine the effect of the discontinuation of ACE inhibitors on the intraoperative hypotension after the induction of general anesthesia [16]. Among the 60 patients, 30 patients were randomized into a group that continued ACE inhibitor therapy till the day of surgery and the other 30 were put into a group that discontinued the therapy one day before the surgery. The study lasted two years, and it showed that there was significant hypotension after the induction of anesthesia. Hence, they concluded that the continuation of ACE inhibitor therapy did cause intraoperative hypotension.

One of the variables that we used for our study was those patients who were on multiple antihypertensive drugs, including ACE inhibitors. Of the total sample size, 31 patients were on antihypertensive therapy apart from ACE inhibitors. These drugs included diuretics, calcium channel blockers, or beta-blockers. Our results showed that 22 out of the 31 (77%) patients on other antihypertensive therapy developed intraoperative hypotension. Although statically insignificant (p>0.05) in this study, these findings can be compared to those of Colson et al. in an earlier study, which showed that the patients who were receiving combination antihypertensive drugs along with ACE inhibitors, were more prone to hypotension [17]. Similarly, Kheterpal et al. concluded that concomitant diuretic therapy with ACE inhibitors can lead to an increased incidence of intraoperative hypotension in an observational study done in 2006 on 9000 patients [18]. Of the 77 patients who did develop hypotension in our study, 41 patients needed the use of vasopressors to correct the hypotension. This can be compared to the double-blinded, randomized control study done by Schirman and Schurman on 100 patients who said that the patients who received ACE inhibitors had to be given vasopressor support more often (17 of the 50 patients ) than patients who didn’t receive the drug (5 out of 50 patients) but still developed hypotension [19].

Another variable that we observed in this study was age. We divided the subjects into three groups that were less than 40 years (n-21) between 41 and 50(n-38) and the third group aged between 51 and 60 (n-56). It was observed that the younger age group was less prone to intraoperative hypotension as compared to the older age group. These findings are in accordance with the conclusion made by Reich et al. who enlisted ages of more than 50 as a predictor of intraoperative hypotension. Reich et al. also said that ASA III patients were more likely to develop intraoperative hypotension although our study found there was no difference in the development of hypotension in the patients labeled ASA II or ASA III [20]. Diabetes was found in 29 (25.22%) patients and 86 patients were non-diabetics. Of these, 19 patients developed intraoperative hypotension. There is scant literature available that predicts diabetes as a risk factor for intraoperative hypotension for hypertensive patients on ACE inhibitors. Reich et al. didn’t find any association of preoperative hypoglycemia with intraoperative hypotension [20]. Furthermore, Kheterpal et al. were unable to confirm insulin-dependent diabetes mellitus as a risk factor for the revised cardiac risk index for patients undergoing general anesthesia in 2009 [21].

In a retrospective review, our study does have its limitations. As this was an observational study, none of the general anesthesia techniques were standardized, which could have affected the results. Other than that, there was no record of patients who had intraoperative difficult airway instrumentation, an implication of fluid therapy, changes in position, or a variety of other potential factors, which might have influenced the results.

## Conclusions

Intraoperative hypotension is more frequent in patients with controlled hypertension on ACE inhibitors although more studies need to be conducted on a larger population in order to determine a more definitive result.
